# Joint effects of diabetes and time-weighted average multidimensional nutritional indices on mortality in patients undergoing peritoneal dialysis: a landmark analysis

**DOI:** 10.3389/fnut.2026.1968835

**Published:** 2026-09-18

**Authors:** Yaohua Hu, Xinyang Li, Ying Yi, Xinyu Gao, Wenpeng Cui

**Affiliations:** Department of Nephrology, The Second Hospital of Jilin University, Changchun, China

**Keywords:** diabetes, longitudinal nutritional assessment, malnutrition, mortality, peritoneal dialysis

## Abstract

**Background:**

Diabetes and malnutrition are critical prognostic indicators for individuals undergoing peritoneal dialysis (PD). Nevertheless, prior research has predominantly focused on initial nutritional assessments, which may not adequately reflect the cumulative burden of long-term nutritional exposure. Consequently, the combined influence of diabetes and longitudinal nutritional changes on mortality risk remains largely unexplored.

**Methods:**

This study retrospectively collected longitudinal follow-up data from patients who underwent PD at the Second Hospital of Jilin University between January 2016 and July 2024, with follow-up continued until July 30, 2025. Utilizing a landmark analysis framework, longitudinal nutritional status was evaluated through Time-Weighted Average (TWA) multidimensional nutritional indices. These indices included the TWA-Controlling Nutritional Status (CONUT) score, TWA-Prognostic Nutritional Index (PNI), and TWA-hemoglobin, albumin, lymphocyte, and platelet (HALP) score. Cox proportional hazards models were employed to determine the associations between diabetes status, longitudinal nutritional indices, and their joint effects with both all-cause and cardiovascular mortality. Furthermore, interaction analyses and restricted cubic spline models were applied to ascertain whether diabetes modulated the relationship between longitudinal nutritional status and mortality risk.

**Results:**

The study cohort comprised 229 patients undergoing PD, with an average age of 48.68 ± 13.42 years; 87 of these patients had diabetes. Among the longitudinal nutritional indices evaluated, the TWA-CONUT score demonstrated the highest predictive accuracy for both all-cause and cardiovascular mortality. Both diabetes and a poorer long-term nutritional status were independently linked to an elevated risk of death. Patients presenting with both diabetes and malnutrition experienced substantially higher risks of all-cause mortality and cardiovascular mortality, with HRs of 5.10 (95% CI, 2.08–12.48) and 6.86 (95% CI, 1.93–24.32), respectively. A statistically significant interaction was observed between diabetes status and the TWA-CONUT score concerning all-cause mortality (*P* for interaction = 0.0037).

**Conclusion:**

The integration of the TWA-CONUT score with diabetes status holds potential for identifying PD patients at an elevated risk of mortality. Specifically, individuals with both diabetes and chronic malnutrition faced significantly increased risks of all-cause and cardiovascular mortality.

## 1 Introduction

Peritoneal dialysis (PD) stands as a crucial kidney replacement therapy for individuals grappling with end-stage kidney disease (1, 2). However, various factors continue to influence long-term outcomes, such as metabolic disturbances, inflammatory burden, and fluctuations in nutritional status (3, 4). Among established prognostic determinants, diabetes is a leading cause of kidney failure and is closely intertwined with malnutrition-related conditions like protein–energy wasting (PEW) (5, 6). Metabolic dysfunction and nutritional derangement may therefore represent a key driver of adverse clinical outcomes in patients undergoing dialysis. Multidimensional nutritional assessment tools, including the Controlling Nutritional Status (CONUT) score and the Prognostic Nutritional Index (PNI), have been extensively used for prognostic stratification (7, 8). However, most previous studies have relied on cross-sectional evaluations, which are typically based on a single baseline measurement (9, 10). Indeed, the nutritional profiles of patients undergoing PD are remarkably dynamic, shaped by fluctuating volume loads, the progressive decline of residual kidney function, and persistent microinflammation (11, 12). A single baseline nutritional indicator may not adequately reflect the cumulative burden of long-term nutritional exposure. Moreover, while the independent adverse effects of diabetes and malnutrition are well recognized, their longitudinal joint effects remain insufficiently characterized. The extent to which diabetes status modifies the mortality risk associated with nutritional deterioration remains largely unexplored in real-world clinical cohorts.

This study introduces a Time-Weighted Average (TWA) multidimensional nutritional index, derived from landmark analysis, to address critical clinical gaps. Its primary objectives are to investigate the independent and combined effects of longitudinal nutritional status and diabetes, and to elucidate their interactive effects on both all-cause and cardiovascular mortality risk in patients with PD. Ultimately, this research seeks to provide longitudinal, evidence-based support for more refined risk stratification within this vulnerable patient population.

## 2 Methods

### 2.1 Study design and participants

This retrospective cohort study screened 518 patients with end-stage renal disease who initiated PD at the Second Hospital of Jilin University between January 1, 2016, and July 31, 2024. Patients were excluded if they had missing baseline data, were < 18 years old, transferred from hemodialysis to PD, had a survival time < 12 months, insufficient nutritional assessments (<2 assessments within 12 months or an assessment interval < 3 months), or missing follow-up data. The detailed exclusion process is shown in Figure 1.

**Figure 1:**
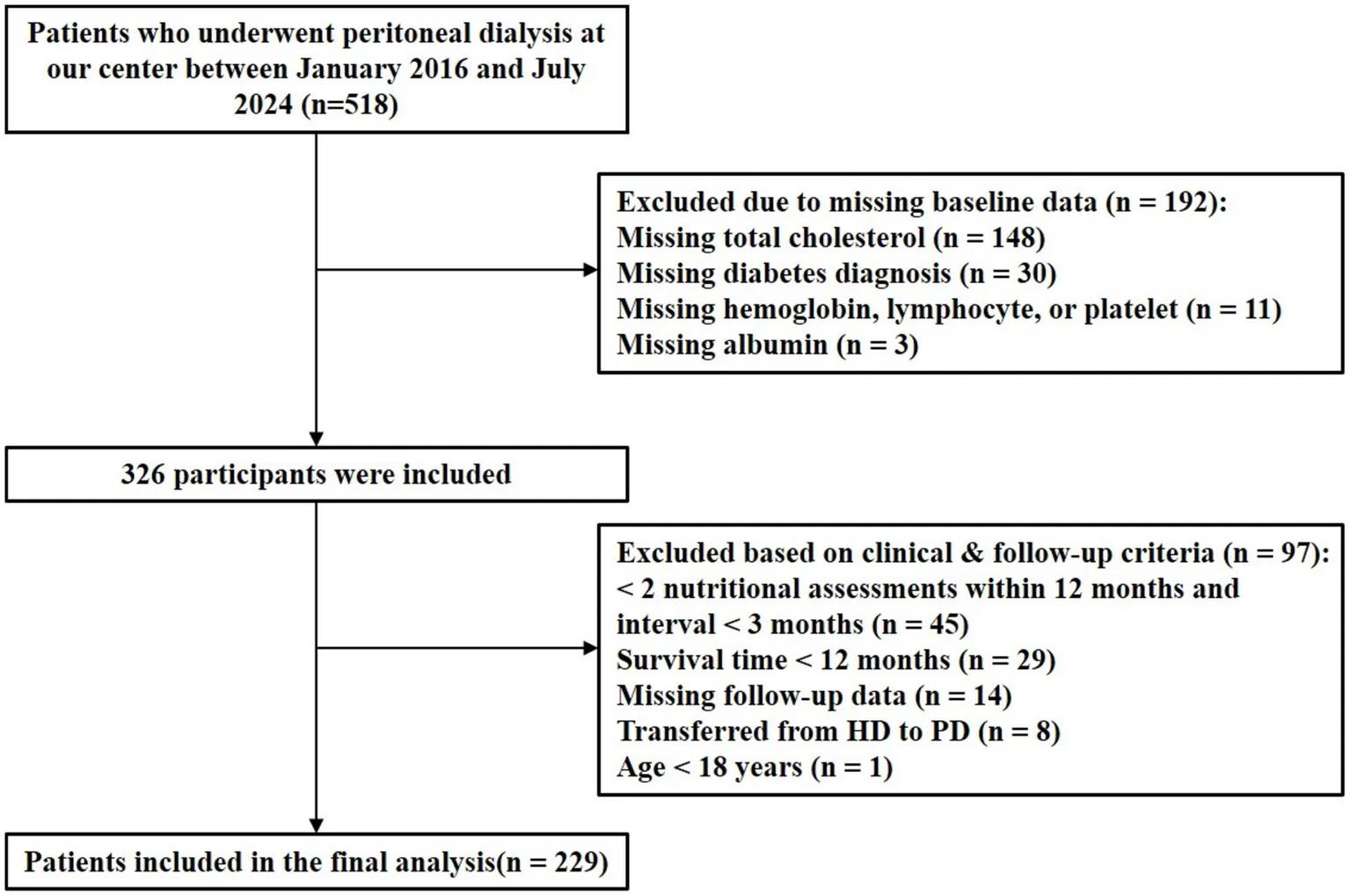
Flowchart of the study population.

Participants were regularly followed until the occurrence of one of the following events: death, transfer to hemodialysis, kidney transplantation, or the study endpoint on July 31, 2025. The Ethics Committee of the Second Hospital of Jilin University approved the study protocol (No. 2025388). Informed consent was not required due to the retrospective design of the study.

### 2.2 Data collection

The longitudinal data were obtained from the follow-up database of the PD Center at the Second Hospital of Jilin University. Baseline clinical variables included demographics (age and sex), lifestyle factors (smoking and alcohol consumption), and comorbidities (diabetes, hypertension, heart disease, and stroke). Concomitant medications collected were ACEIs/ARBs, CCBs, diuretics, and α- or β-adrenergic blockers. Clinical and laboratory measurements comprised BMI, hemoglobin, serum albumin, total cholesterol, triglycerides, lymphocyte count, platelet count, and estimated glomerular filtration rate (eGFR).

### 2.3 Definitions of variables

Diabetes status was determined by a documented clinical diagnosis found in the patients’ electronic medical records. In this study, the TWA index was used to summarize the average longitudinal nutritional status during the landmark period. Specifically, TWA values for three nutritional scores were calculated using the trapezoidal rule, incorporating serial measurements obtained within a 12-month landmark window after dialysis initiation. The PNI was calculated as: PNI = serum albumin (g/L) + 5 × lymphocyte count (×10^9^/L). The hemoglobin, albumin, lymphocyte, and platelet (HALP) score was defined as: HALP = hemoglobin (g/L) × albumin (g/L) × lymphocyte count (×10^9^/L) / platelet count (×10^9^/L). The CONUT score was determined using a structured scoring system that incorporates serum albumin, total cholesterol, and lymphocyte count (detailed scoring criteria are provided in Supplementary Table S1). To assess the distinct and combined effects of diabetes and nutritional status, patients were stratified into four exclusive groups. This categorization was based on their diabetes status and TWA nutritional status (good vs. poor nutrition). The groups were: (1) no diabetes + good nutrition, which served as the reference group; (2) no diabetes + poor nutrition; (3) diabetes + good nutrition; and (4) diabetes + poor nutrition. The main endpoint for this study was all-cause mortality, while cardiovascular mortality was the secondary outcome. All patients were followed from the index date (marking the end of the 12-month landmark window) until their death, or censored if they initiated maintenance hemodialysis, underwent renal transplantation, or reached the administrative end of the study follow-up on July 31, 2025, whichever occurred first.

### 2.4 Handling of missing variables

Supplementary Table S2 summarizes the distribution of missing baseline data. In this dataset, the estimated glomerular filtration rate (eGFR) was unavailable for six cases, representing 2.6% of the study population. Given the low proportion of missing data and the non-normal distribution of eGFR, missing values were handled using median imputation.

### 2.5 Statistical analysis

Continuous variables were summarized as mean ± SD or median (interquartile range), as appropriate, and compared using Student’s *t*-test or the Mann–Whitney *U* test. Categorical variables were expressed as counts and percentages and analyzed using the chi-square or Fisher’s exact test, as appropriate.

To identify the optimal longitudinal nutritional index for clinical risk prediction, we evaluated and compared the incremental predictive value of adding TWA-PNI, TWA-CONUT, or TWA-HALP to a baseline clinical model. This model included age, sex, smoking status, alcohol consumption, hypertension, heart disease, stroke, diabetes, and eGFR. Model discrimination was evaluated using Harrell’s concordance index (C-index), the Akaike information criterion (AIC), and the area under the 3-year time-dependent receiver operating characteristic curve (time-dependent AUC). We used the likelihood ratio test (LRT) to evaluate model improvement. TWA-CONUT was selected as the optimal nutritional marker for the joint analysis. Patients were then classified into four mutually exclusive phenotype groups based on their diabetes status and TWA-CONUT-defined nutritional status (favorable vs. poor). Kaplan–Meier methods were used to estimate cumulative survival curves for all-cause and cardiovascular mortality across these four joint groups. Finally, multivariable Cox proportional hazards regression models were constructed to estimate HR and 95% CI for the independent and joint associations. Three sequentially adjusted models were fitted. Restricted cubic spline (RCS) analysis with three knots was performed within the Cox regression framework to evaluate the continuous association between TWA-CONUT and mortality risk, stratified by diabetes status. All statistical tests were two-sided, and a *p*-value < 0.05 was considered statistically significant. Statistical analyses and data visualizations were performed using R software version 4.4.1 and GraphPad Prism version 10.0, respectively.

## 3 Results

### 3.1 Baseline characteristics

This study included 229 patients with PD, 87 of whom also had diabetes. To evaluate potential selection bias associated with the landmark design, we compared the baseline characteristics of the 229 included patients with those of the 289 excluded patients (Supplementary Table S3). No significant differences were observed in age, sex, comorbidities, or baseline laboratory measurements between the two groups. However, the included cohort had a lower proportion of smokers (*p* = 0.049) and patients reporting alcohol consumption (*p* < 0.001), as well as a higher rate of CCB use (*p* = 0.014). Table 1 presents the baseline characteristics of these patients, stratified by their diabetes status. Patients with diabetes were significantly older than those without (52.68 ± 12.31 years vs. 46.23 ± 13.52 years, *p* < 0.001) and had a higher proportion of men (72.4% vs. 53.5%, *p* = 0.004).

**Table 1:** Baseline characteristics of the study population according to diabetes status.

Characteristics	Total (*n* = 229)	Non-diabetes (*n* = 142)	Diabetes (*n* = 87)	*p-*value
Age, years	48.68 ± 13.42	46.23 ± 13.52	52.68 ± 12.31	<0.001
Male, *n* (%)	139 (60.7%)	76 (53.5%)	63 (72.4%)	0.004
Smoking, *n* (%)	35 (15.3%)	18 (12.7%)	17 (19.5%)	0.161
Alcohol consumption, *n* (%)	13 (5.7%)	5 (3.5%)	8 (9.2%)	0.072
Comorbidities				
Hypertension, *n* (%)	211 (92.1%)	128 (90.1%)	83 (95.4%)	0.151
Heart disease, *n* (%)	80 (34.9%)	43 (30.3%)	37 (42.5%)	0.059
Stroke, *n* (%)	30 (13.1%)	14 (9.9%)	16 (18.4%)	0.063
Clinical and Laboratory Measures				
BMI, kg/m^2^	23.90 ± 3.97	22.61 ± 3.45	26.02 ± 3.89	<0.001
Hemoglobin, g/L	91.16 ± 22.53	90.05 ± 23.25	92.97 ± 21.31	0.343
Albumin, g/L	36.39 ± 5.31	37.70 ± 5.04	34.24 ± 5.08	<0.001
Total cholesterol, mmol/L	4.57 ± 1.16	4.50 ± 1.07	4.69 ± 1.28	0.233
Triglycerides, mmol/L	1.61 (1.18–2.17)	1.52 (1.16–2.11)	1.63 (1.33–2.27)	0.101
Lymphocyte count, ×10^9^/L	1.20 (0.90–1.60)	1.20 (0.83–1.50)	1.30 (0.90–1.75)	0.104
Platelet count, ×10^9^/L	184.00 (143.00–228.70)	174.50 (136.25–219.00)	190.00 (158.00–239.50)	0.032
eGFR, mL/min/1.73m^2^	5.95 (4.48–7.85)	5.38 (4.14–7.36)	6.98 (5.50–9.07)	<0.001
Medications				
ARB/ACEI, *n* (%)	66 (28.8%)	39 (27.5%)	27 (31.0%)	0.563
CCB, *n* (%)	158 (69.0%)	93 (65.5%)	65 (74.7%)	0.143
Diuretic, *n* (%)	18 (7.9%)	8 (5.6%)	10 (11.5%)	0.110
Beta-blockers, *n* (%)	105 (45.9%)	58 (40.9%)	47 (54.0%)	0.052
Longitudinal Nutritional Indices				
TWA-PNI	44.29 ± 5.48	45.20 ± 5.45	42.81 ± 5.23	0.001
TWA-HALP	26.58 (19.51–35.49)	27.23 (20.26–35.52)	26.06 (18.06–35.16)	0.369
TWA-CONUT	2.25 (1.25–3.50)	2.00 (1.19–3.36)	2.67 (1.50–3.86)	0.048

BMI, body mass index; eGFR, estimated glomerular filtration rate; ARB/ACEI, angiotensin receptor blockers/angiotensin-converting enzyme inhibitors; CCB, calcium channel blockers; TWA-PNI, Time-Weighted Average prognostic nutritional index; TWA-HALP, Time-Weighted Average hemoglobin, albumin, lymphocyte, and platelet score; TWA-CONUT, Time-Weighted Average controlling nutritional status score.

Patients with diabetes had a significantly higher BMI (*p* < 0.001) and eGFR (*p* < 0.001), but lower serum albumin (*p* < 0.001). Longitudinal nutritional assessment showed poorer nutritional status in diabetic patients, with significantly lower TWA-PNI (*p* = 0.001) and higher TWA-CONUT scores (*p* = 0.048). No significant differences were found in lifestyle, comorbidities, blood cell counts, or medication use (all *p* > 0.05).

### 3.2 Incremental predictive value of longitudinal nutritional scores

Supplementary Table S4 presents the frequency distribution of concurrent laboratory measurements utilized to calculate TWA nutritional indices over the 12-month landmark period. Patients had a median of 3 measurements (IQR, 2–4). To identify the most effective longitudinal nutritional index for clinical risk prediction, each TWA nutritional index was progressively incorporated into a baseline model that included conventional risk factors (Table 2).

**Table 2:** Incremental predictive value of longitudinal nutritional scores for mortality outcomes.

Outcomes and models	Harrell’s C-index (95% CI)	*P-*value	AIC	LRT (χ^2^)	*P-*value	Time-dependent AUC (3-year)
All-cause mortality						
Basic model	0.768 (0.708–0.828)	Ref.	538.4	Ref.	Ref.	0.801
Basic model + TWA-PNI	0.808 (0.756–0.860)	0.036	525.3	15.0	<0.001	0.855
Basic model + TWA-CONUT	0.818 (0.766–0.869)	0.021	514.5	25.9	<0.001	0.861
Basic model + TWA-HALP	0.787 (0.731–0.843)	0.063	535.1	5.2	0.022	0.822
Cardiovascular mortality						
Basic model	0.800 (0.719–0.882)	Ref.	241.5	Ref.	Ref.	0.804
Basic model + TWA-PNI	0.827 (0.768–0.886)	0.273	239.6	3.9	0.048	0.864
Basic model + TWA-CONUT	0.838 (0.777–0.899)	0.234	237.7	5.8	0.016	0.889
Basic model + TWA-HALP	0.811 (0.736–0.886)	0.271	241.0	2.5	0.110	0.833

CI, confidence interval; Ref, reference; AIC, Akaike information criterion; LRT, likelihood ratio test; AUC, area under the curve; TWA-PNI, Time-Weighted Average prognostic nutritional index; TWA-CONUT, Time-Weighted Average controlling nutritional status score; TWA-HALP, Time-Weighted Average hemoglobin, albumin, lymphocyte, and platelet score. The basic model included age, sex, hypertension, diabetes, heart disease, stroke, smoking, alcohol consumption, and eGFR.

For predicting all-cause mortality, the integration of either TWA-CONUT or TWA-PNI into the baseline model (C-index: 0.768; 3-year AUC: 0.801) led to a significant enhancement in both model discrimination and goodness of fit. The most substantial improvement was observed with TWA-CONUT, elevating the C-index to 0.818 (95% CI: 0.766–0.869, *p* = 0.021) and the 3-year AUC to 0.861, concurrently achieving a significant reduction in the AIC (LRT *p* < 0.001). While the inclusion of TWA-HALP did not yield a statistically significant improvement in the C-index (*p* = 0.063), it did contribute to an improved model fit (LRT *p* = 0.022). Regarding cardiovascular mortality, the baseline model exhibited a C-index of 0.800 and a 3-year AUC of 0.804. Although neither TWA-CONUT nor TWA-PNI significantly increased Harrell’s C-index (both *p* > 0.05), both indices notably improved model fit (both LRT *p* < 0.05) and boosted the 3-year AUC to 0.889 and 0.864, respectively. Conversely, the addition of TWA-HALP did not result in a significant enhancement of predictive performance for cardiovascular mortality (LRT *p* = 0.110).

### 3.3 Independent and joint effects of diabetes and nutritional status on mortality

During the follow-up period, a total of 61 all-cause deaths and 29 cardiovascular deaths were recorded. Kaplan–Meier survival curves revealed highly significant differences in cumulative survival for both all-cause and cardiovascular mortality among groups categorized by diabetes and nutritional status, as determined by TWA-CONUT (both log-rank *p* < 0.001) (Figure 2). Patients presenting with both diabetes and poor nutritional status exhibited the lowest survival probability.

**Figure 2:**
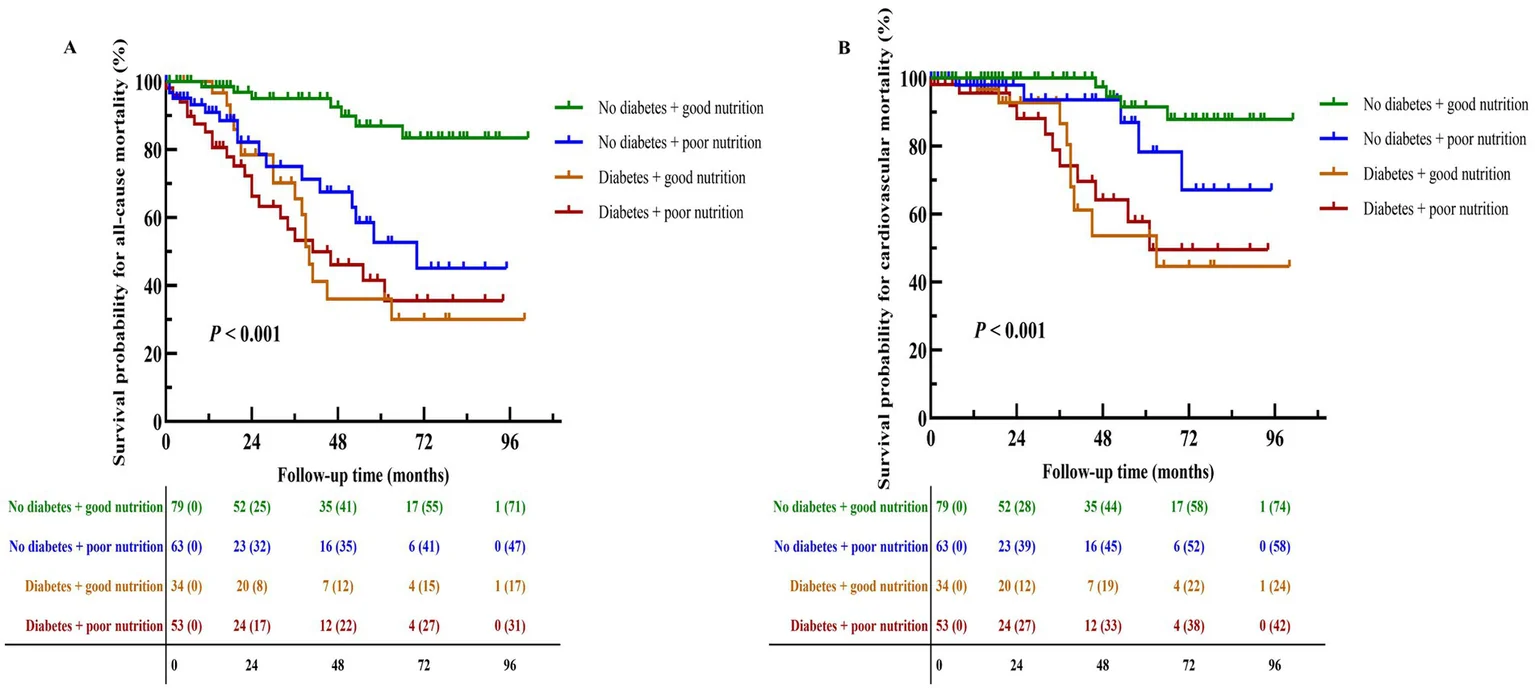
Kaplan–Meier survival curves for mortality outcomes stratified by the joint effect of diabetes and longitudinal nutritional status. **(A)** All-cause mortality. **(B)** Cardiovascular mortality.

Multivariable Cox regression analyses were conducted to evaluate the independent effects of diabetes and poor nutritional status (Table 3). Both factors were significantly associated with an elevated risk of all-cause mortality after comprehensive adjustment for confounders (Model 3; HR = 2.03 and 2.25, respectively; both *p* < 0.05). For cardiovascular mortality, diabetes remained a robust independent predictor (HR = 4.08, *p* = 0.002), while the independent effect of poor nutritional status did not achieve statistical significance (*p* = 0.132).

**Table 3:** Independent and joint associations of nutrition status and diabetes with all-cause and cardiovascular mortality.

Variables	Model 1 HR (95% CI)	*P-*value	Model 2 HR (95% CI)	*P-*value	Model 3 HR (95% CI)	*P-*value
All-cause mortality						
Nutrition status						
Good nutrition	1.00 (Ref)	–	1.00 (Ref)	–	1.00 (Ref)	–
Poor nutrition	2.38 (1.41–4.00)	0.001	2.03 (1.20–3.45)	0.008	2.25 (1.29–3.91)	0.004
Diabetes status						
No	1.00 (Ref)	–	1.00 (Ref)	–	1.00 (Ref)	–
Yes	3.24 (1.93–5.46)	<0.001	2.32 (1.35–3.99)	0.002	2.03 (1.16–3.55)	0.013
Joint effect						
No diabetes + Good nutrition	1.00 (Ref)	–	1.00 (Ref)	–	1.00 (Ref)	–
No diabetes + Poor nutrition	4.60 (1.89–11.21)	0.001	3.95 (1.62–9.65)	0.003	4.41 (1.77–11.02)	0.002
Diabetes + Good nutrition	6.85 (2.81–16.71)	<0.001	4.64 (1.87–11.53)	0.001	4.08 (1.59–10.48)	0.004
Diabetes + Poor nutrition	7.39 (3.15–17.36)	<0.001	5.23 (2.18–12.58)	<0.001	5.10 (2.08–12.48)	<0.001
Cardiovascular mortality						
Nutrition status						
Good nutrition	1.00 (Ref)	–	1.00 (Ref)	–	1.00 (Ref)	–
Poor nutrition	1.92 (0.92–4.01)	0.081	1.44 (0.68–3.04)	0.341	1.84 (0.83–4.08)	0.132
Diabetes status						
No	1.00 (Ref)	–	1.00 (Ref)	–	1.00 (Ref)	–
Yes	4.92 (2.23–10.86)	<0.001	3.45 (1.53–7.79)	0.003	4.08 (1.69–9.87)	0.002
Joint effect						
No diabetes + Good nutrition	1.00 (Ref)	–	1.00 (Ref)	–	1.00 (Ref)	–
No diabetes + Poor nutrition	2.79 (0.75–10.42)	0.126	2.30 (0.61–8.63)	0.216	3.25 (0.82–12.85)	0.093
Diabetes + Good nutrition	7.94 (2.43–25.90)	<0.001	5.78 (1.74–19.13)	0.004	6.91 (1.93–24.79)	0.003
Diabetes + Poor nutrition	7.47 (2.37–23.57)	<0.001	4.61 (1.41–15.02)	0.011	6.86 (1.93–24.32)	0.003

Model 1 was unadjusted.

Model 2 was adjusted for age and sex.

Model 3 was fully adjusted for age, sex, hypertension, heart disease, stroke, smoking, alcohol consumption, and eGFR. The joint effect variable categorized patients into four mutually exclusive groups based on the presence of diabetes and nutritional status.

The joint-effect analysis demonstrated a particularly pronounced combined adverse impact of diabetes and poor nutritional status. When using patients without diabetes and with favorable nutritional status as the reference group, the risk of all-cause mortality progressively increased with the accumulation of risk factors in the fully adjusted model (Model 3). Specifically, patients with both diabetes and poor nutritional status displayed the highest risk of all-cause mortality, with an HR of 5.10 (95% CI: 2.08–12.48, *p* < 0.001). Concerning cardiovascular mortality, diabetes status emerged as the dominant risk driver; patients with diabetes and poor nutritional status experienced a similarly pronounced elevation in risk (HR: 6.86, 95% CI: 1.93–24.32, *p* = 0.003).

### 3.4 Interaction and sensitivity analyses

Restricted cubic spline (RCS) analyses were employed to elucidate the continuous dose–response relationship between TWA-CONUT and mortality risk, stratified by diabetes status (Figure 3). Among individuals without diabetes, TWA-CONUT exhibited a monotonically positive association with all-cause mortality risk. Conversely, a nonlinear relationship was observed in participants with diabetes. The significant interaction test (*p* = 0.0037) for interaction confirmed diabetes status as a strong modifier of the association between TWA-CONUT and all-cause mortality. Regarding cardiovascular mortality, an increased risk of death correlated with deteriorating nutritional status in both groups, and no significant interaction was detected (*P* for interaction = 0.2970).

**Figure 3:**
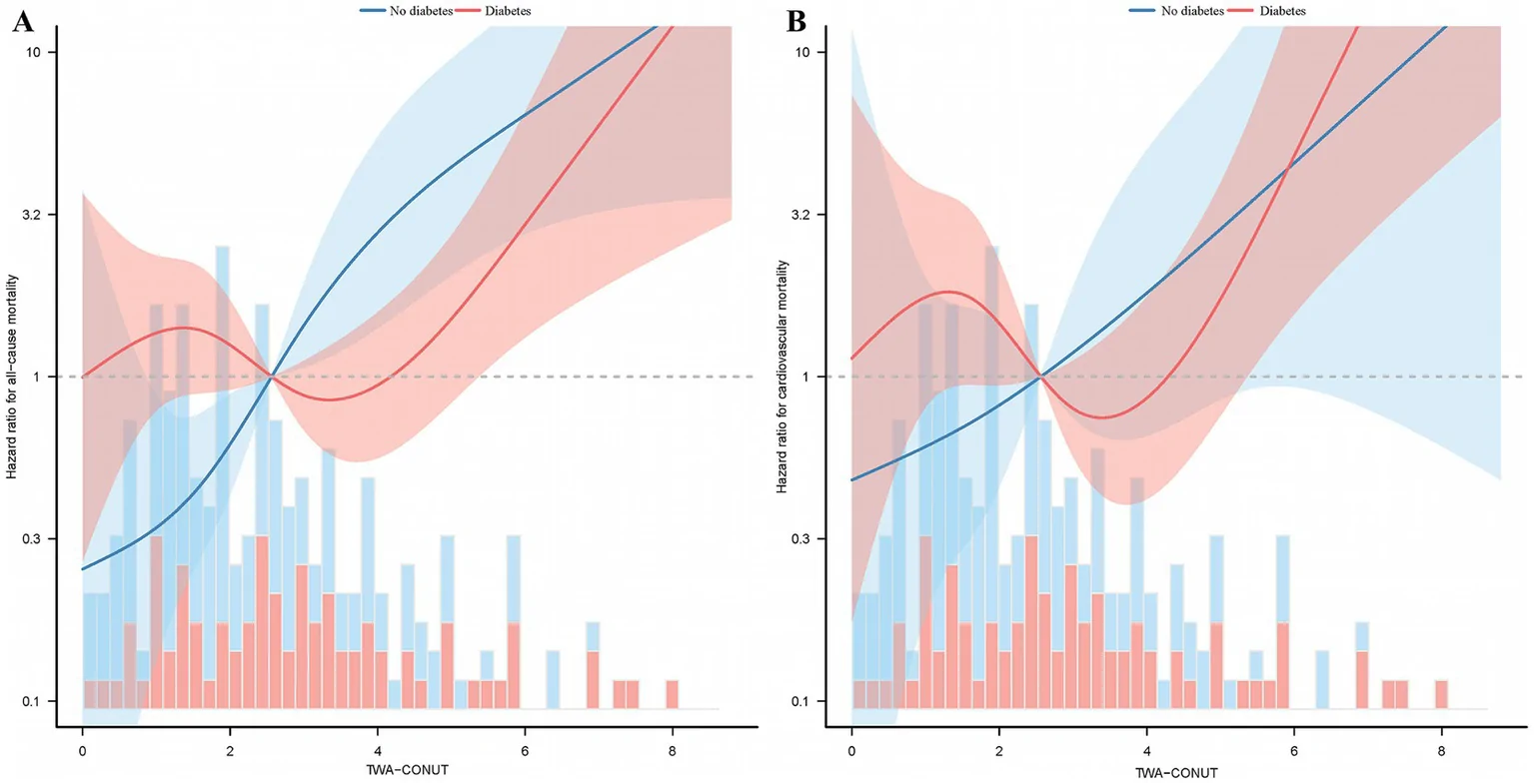
Restricted cubic spline curves showing the continuous dose–response association of TWA-CONUT with mortality risks, stratified by diabetes status. **(A)** All-cause mortality. **(B)** Cardiovascular mortality. The *p* values for interaction between TWA-CONUT and diabetes status were 0.0037 for all-cause mortality and 0.2970 for cardiovascular mortality.

Sensitivity analyses were also performed to compare the predictive performance of TWA nutritional indices against single baseline measurements (Supplementary Table S5). The TWA-CONUT model demonstrated higher absolute C-index values than the baseline CONUT model for both all-cause mortality (0.818 vs. 0.789; ΔC-index = 0.029) and cardiovascular mortality (0.838 vs. 0.819; ΔC-index = 0.018). However, these observed improvements did not achieve statistical significance (*p* = 0.076 and 0.359, respectively).

## 4 Discussion

This study systematically compared several longitudinal nutritional indices, identifying TWA-CONUT as the most informative nutritional predictor. When incorporated into a conventional clinical risk model, TWA-CONUT significantly enhanced the predictive value for both all-cause and cardiovascular mortality. Joint-effect analyses further revealed that patients with both diabetes and malnutrition experienced markedly higher risks of all-cause and cardiovascular mortality, with fully adjusted hazard ratios of 5.10 and 6.86, respectively. Furthermore, restricted cubic spline analyses and interaction testing demonstrated a significant interaction between diabetes status and TWA-CONUT concerning all-cause mortality. These findings underscore the prognostic relevance of cumulative nutritional exposure in patients undergoing PD and suggest that integrating longitudinal nutritional assessment with diabetes status could improve the identification of high-risk patients within this population.

Our findings expand upon prior research concerning nutritional monitoring in PD cohorts. For instance, Zhou et al. previously demonstrated a significant association between an elevated CONUT score and an increased risk of all-cause mortality among patients undergoing PD (13), and similar findings were reported by Qiu et al. (14). In addition, a multicenter cohort study from China, which included 404 patients undergoing PD, found that a lower PNI was associated with a higher peritoneal transport status, potentially contributing to an increased risk of mortality (15). These observations align with our findings. However, previous studies predicting mortality often relied on baseline nutritional indicators and therefore did not account for cumulative nutritional exposure over time. In contrast, this study employed a landmark analysis framework and incorporated TWA multidimensional indices to summarize the cumulative nutritional exposure over the landmark period. Consistent with our results, two prior studies from China and Japan have also identified diabetes and malnutrition as independent risk factors for survival in patients undergoing PD (16, 17). Building on this evidence, we further examined the joint effect of diabetes and poor nutritional status from a longitudinal perspective. In our cohort, the coexistence of these conditions was associated with 5.10-fold and 6.86-fold higher risks of all-cause and cardiovascular mortality, respectively. The interaction between diabetes and poor nutritional status was further supported by the RCS model. These findings offer a more refined stratification approach for identifying this high-risk subgroup.

Diabetes and sustained malnutrition were significantly associated with increased mortality in patients undergoing PD, an observation that may be linked to the malnutrition–inflammation–atherosclerosis (MIA) syndrome (18). Diabetes stands as an independent risk factor, potentially accelerating atherosclerosis through its impact on endothelial dysfunction, chronic systemic inflammation, and metabolic dysregulation, ultimately elevating susceptibility to cardiovascular events (19). Malnutrition, characterized by protein-energy wasting, is intricately linked to compromised immune function and an elevated inflammatory state. This connection positions malnutrition as a core element of the MIA syndrome (20). In PD, long-term exposure to glucose-based dialysate may aggravate local and systemic inflammation by increasing peritoneal solute transport rate (21, 22); together with the decline in residual kidney function and peritoneal protein loss, these processes may further disrupt nutritional and immune homeostasis (23). Diabetes can exacerbate the pathological effects of malnutrition through several mechanisms. Hyperglycemia, for instance, may intensify oxidative metabolic stress and microvascular injury. Concurrently, diminished nutritional reserves can compromise the body’s capacity to counteract dialysis-related metabolic stress. This confluence of factors contributes to a detrimental cycle of metabolic, nutritional, and inflammatory disturbances (24, 25). The nonlinear association between TWA-CONUT and all-cause mortality observed in the diabetic subgroup may partly relate to the individual components of the CONUT score, particularly TC. Lower TWA-CONUT scores generally correspond to more favorable cholesterol, albumin, and lymphocyte profiles. For diabetic patients undergoing PD, elevated cholesterol is frequently associated with dyslipidemia, atherosclerosis, and increased cardiovascular risk, which may attenuate the survival advantage of adequate nutritional reserves (26). Conversely, higher TWA-CONUT scores reflect PEW and systemic inflammation. In the presence of diabetes, PEW exacerbates oxidative stress and endothelial dysfunction, leading to a marked increase in mortality (27). Thus, the observed nonlinear association suggests that both the metabolic burden at lower scores and malnutrition at higher scores contribute to adverse outcomes in this specific population. Moreover, previous studies have reported that nutritional education or nutritional intervention can improve nutritional and metabolic parameters in patients with advanced chronic kidney disease or those undergoing dialysis and may be associated with reduced hospitalization or improved long-term outcomes (28, 29). Therefore, regular nutritional assessment and individualized dietary management may be particularly important for PD patients with diabetes.

Furthermore, although longitudinal indices are designed to reflect cumulative nutritional exposure, the sensitivity analysis showed that the higher C-index of TWA-CONUT compared with baseline CONUT did not reach statistical significance. This indicates a numerical improvement rather than a statistically confirmed superiority. Several factors may explain: First, a single baseline CONUT assessment may already capture a substantial proportion of the prognostic information, leaving a narrow margin for incremental value. Second, the relatively small sample size and the limited number of repeated measurements during the landmark window may have reduced the statistical power to detect a significant difference. Therefore, the incremental benefit of TWA nutritional assessment over baseline screening requires further verification in larger cohorts with higher-frequency data collection.

This study boasts several strengths. Firstly, its longitudinal design, coupled with landmark analysis, facilitated a more comprehensive assessment of cumulative nutritional exposure in patients undergoing PD compared to a singular baseline measurement. Secondly, through a systematic comparison of various TWA multidimensional nutritional indices, this study identified the relative advantage of TWA-CONUT in predicting mortality risk. Thirdly, we further assessed the combined effect and interaction between diabetes and long-term malnutrition, offering more precise evidence for identifying subgroups within the PD population at an elevated risk of mortality. Clinically, these findings suggest that nutritional status should be carefully monitored and optimized, particularly in PD patients with diabetes, as persistent malnutrition may further worsen prognosis.

This study has several limitations. First, the single-center, retrospective design inherently restricts the ability to draw causal inferences and limits the generalizability of the findings. Second, the landmark design may have introduced selection bias, although the included and excluded patients were comparable in terms of major baseline demographic characteristics, comorbidities, and laboratory parameters. Consequently, these findings primarily apply to patients who survived the first year of PD with adequate longitudinal monitoring. Third, despite adjusting the models for numerous clinical factors, the complete exclusion of residual confounding remains a challenge. Fourth, the relatively small sample size and limited number of cardiovascular deaths reduced the statistical power of the secondary outcome analysis. Consequently, the absence of a statistically significant interaction between diabetes status and TWA-CONUT for cardiovascular mortality may reflect a false-negative finding due to insufficient statistical power. This result should therefore be interpreted with caution. Finally, the assessment of nutritional status relied predominantly on laboratory-derived indices, lacking direct measurements such as dietary intake, body composition, or inflammatory biomarkers. Consequently, these findings necessitate validation through prospective, multicenter studies.

## 5 Conclusion

This study demonstrated that, in patients undergoing PD, among the evaluated longitudinal nutritional indices, TWA-CONUT exhibited the highest predictive performance for both all-cause and cardiovascular mortality. Diabetes and long-term malnutrition were independently associated with an elevated risk of death, and their co-occurrence further amplified this risk. Integrating diabetes status with longitudinal nutritional assessment could enhance the accurate identification of high-risk patients within the PD population and inform individualized nutritional monitoring and risk management strategies.

## Data Availability

The raw data supporting the conclusions of this article will be made available by the authors, without undue reservation.

## References

[1] Hu Y-H Liu Y-L Meng L-F Zhang Y-X Cui W-P. Selection of dialysis methods for end-stage kidney disease patients with diabetes. World J Diabetes. (2024) 15:1862–73. doi: 10.4239/wjd.v15.i9.1862 PMC11372645 39280188

[2] Corbett RW Beckwith H Lucisano G Brown EA. Delivering person-centered peritoneal Dialysis. Clin J Am Soc Nephrol. (2023) 19:377–84. doi: 10.2215/CJN.0000000000000281, 37611155 PMC10937028

[3] Xu Y Zhong Z Li Y Li Z Zhou Y Li Z . Interaction effect between fasting plasma glucose and lipid profiles on mortality of peritoneal dialysis patients. Clin Kidney J. (2022) 16:727–34. doi: 10.1093/ckj/sfac266, 37007694 PMC10061421

[4] Xu P Zhang L Hao L Li J Jiang C. Protecting peritoneal dialysis patients: evaluation and prevention strategies for noninfectious risk factors. Ren Fail. (2026) 48:2667650. doi: 10.1080/0886022X.2026.2667650, 42124464 PMC13173579

[5] Khachroumi N Boyer A Lanot A Ficheux M Bechade C Lobbedez T. Outcomes of diabetic patients undergoing peritoneal dialysis in the French language peritoneal dialysis registry. Clin Kidney J. (2025) 19:sfaf382. doi: 10.1093/ckj/sfaf382, 41522690 PMC12783895

[6] Hoppe K Schwermer K Dopierała M Kałużna M Hoppe A Chou JT-T . Can overnutrition lead to wasting?-the paradox of diabetes mellitus in end-stage renal disease treated with maintenance hemodialysis. Nutrients. (2022) 14:247. doi: 10.3390/nu14020247, 35057428 PMC8779228

[7] Li X Zhou X Li K Pu L He X Liu X . Enhanced prognostic value of a composite nutritional-inflammatory index (P-CONUT) for predicting mortality risk in patients initiating peritoneal dialysis. PLoS One. (2025) 20:e0323318. doi: 10.1371/journal.pone.0323318, 40403009 PMC12097555

[8] Yang Y Xu Y Zhang P Zhou H Yang M Xiang L. Predictive value of objective nutritional indexes in technique failure in peritoneal Dialysis patients. J Ren Nutr. (2021) 32:605–12. doi: 10.1053/j.jrn.2021.09.005, 34776339

[9] Yang Y Zhou H Zhang P Chao W Zou Y Yang M. Evaluation of objective nutritional indexes as predictors of worse outcomes in peritoneal dialysis patients. Nutrition. (2020) 79-80:110963. doi: 10.1016/j.nut.2020.110963, 33011471

[10] Lin W Wu J Zhan X Wen Y Wang X Feng X . A combined nutritional index and mortality in patients with peritoneal dialysis. Ren Fail. (2025) 47:2541069. doi: 10.1080/0886022X.2025.2541069, 40785303 PMC12340938

[11] Chen T Liu F Zou Y Yang M. C-reactive protein to albumin ratio predicts future volume overload in peritoneal dialysis patients: a retrospective cohort study. Biomark Med. (2026) 20:191–8. doi: 10.1080/17520363.2026.2634065, 41728829 PMC12947583

[12] Yeter HH. Preserving residual kidney function in peritoneal dialysis: from conventional approaches to contemporary practice. Clin Kidney J. (2026) 19:sfag131. doi: 10.1093/ckj/sfag131, 42137551 PMC13170695

[13] Zhou H Chao W Cui L Li M Zou Y Yang M. Controlling nutritional status (CONUT) score as immune-nutritional predictor of outcomes in patients undergoing peritoneal dialysis. Clin Nutr. (2019) 39:2564–70. doi: 10.1016/j.clnu.2019.11.018, 31787366

[14] Qiu W Chu R Ma Y Xue J Xu Q Wen Y . Effectiveness of CONUT and NRI as nutritional risk screening tools in peritoneal dialysis: a multicenter study. Front Nutr. (2025) 12:1544338. doi: 10.3389/fnut.2025.1544338, 40416383 PMC12098072

[15] Huang Y Zhang X Tang X Tang L Shang S Wang X . A low prognostic nutritional index is a risk factor for high peritoneal transport status in patients undergoing peritoneal Dialysis. J Ren Nutr. (2022) 33:201–7. doi: 10.1053/j.jrn.2022.03.007, 35367359

[16] Cao Q Shi Y Liu X Yang F Li X Li Z. Analysis of factors influencing vascular calcification in peritoneal dialysis patients and their impact on long-term prognosis. BMC Nephrol. (2024) 25:157. doi: 10.1186/s12882-024-03582-2, 38714960 PMC11075370

[17] Hori S Tomizawa M Inoue K Yoneda T Tachibana A Miyamoto T . Impact of nutrition and inflammation indicators on peritoneal dialysis initiation and management. Clin Exp Nephrol. (2023) 28:349–58. doi: 10.1007/s10157-023-02424-z, 37995061

[18] Chan GC-K Fung WW-S Szeto C-C Ng JK-C. From MIA to FIFA: the vicious matrix of frailty, inflammation, fluid overload and atherosclerosis in peritoneal dialysis. Nephrology (Carlton). (2023) 28:215–26. doi: 10.1111/nep.14150, 36807408

[19] Zhou M Huang H Zhou C. Impact of immune marker-based microinflammation on dialysis efficacy in diabetic nephropathy patients: development of an early prediction model. Clin Nephrol. (2026) 105:308–17. doi: 10.5414/CN111843 41793703

[20] Zhou H Yao C Song K Zhao S Yuan Y Chen X . Machine learning-based prediction of early-onset peritoneal dialysis-associated peritonitis: the role of the CONUT score. Front Nutr. (2025) 12:1681169. doi: 10.3389/fnut.2025.1681169, 41459072 PMC12739878

[21] Herzog R Daniel-Fischer L Eibensteiner F Sobieszek IJ Unterwurzacher M Sacnun JM . Local immunometabolic intervention with alanyl-glutamine resolves peritoneal dialysis-induced immune cell dysfunction. Kidney Int Rep. (2026) 11:106342. doi: 10.1016/j.ekir.2026.106342, 42006205 PMC13087715

[22] Elphick EH Zavvos V Belcher J Topley N Chess JA Holmes CJ . The role of peritoneal Interleukin-6 in predicting patient survival on peritoneal Dialysis. Kidney Int Reports. (2025) 10:3164–73. doi: 10.1016/j.ekir.2025.06.049, 40980662 PMC12446933

[23] Weng N Yi F Liao H Chen H Zhang M Sun Y . Clinical relevance of malnutrition-inflammation score in detecting peritoneal sclerosis among long-term peritoneal dialysis patients. J Ren Nutr. (2026) 26:S1051-2276(26)00095-6. doi: 10.1053/j.jrn.2026.05.001, 42202954

[24] Lambie M Bonomini M Davies SJ Accili D Arduini A Zammit V. Insulin resistance in cardiovascular disease, uremia, and peritoneal dialysis. Trends Endocrinol Metab. (2021) 32:721–30. doi: 10.1016/j.tem.2021.06.001, 34266706 PMC8893168

[25] Lu X Xie Q Pan X Zhang R Zhang X Peng G . Type 2 diabetes mellitus in adults: pathogenesis, prevention and therapy. Signal Transduct Target Ther. (2024) 9:262. doi: 10.1038/s41392-024-01951-9, 39353925 PMC11445387

[26] Chen L Chen S Bai X Su M He L Li G . Low-density lipoprotein cholesterol, cardiovascular disease risk, and mortality in China. JAMA Netw Open. (2024) 7:e2422558. doi: 10.1001/jamanetworkopen.2024.22558, 39023892 PMC11258592

[27] Prokopidis K Chen Y Liu Y Zhong Z Morwani-Mangnani J Cuthbertson DJ . Assessing the link of malnutrition with diabetes and mortality risk in heart failure patients. ESC Heart Fail. (2025) 12:3103–13. doi: 10.1002/ehf2.15263, 40074368 PMC12287868

[28] Vaz de Melo Ribeiro P Miranda Hermsdorff HH Balbino KP de Paula Santos Epifânio A de Paula Jorge M Bandeira Moreira AV. Effect of a nutritional intervention, based on transtheoretical model, on metabolic markers and food consumption of individuals undergoing hemodialysis. J Ren Nutr. (2020) 30:430–9. doi: 10.1053/j.jrn.2019.12.004, 32037084

[29] Pérez-Torres A González García ME Ossorio-González M Álvarez García L Bajo MA Del Peso G . The effect of nutritional interventions on long-term patient survival in advanced chronic kidney disease. Nutrients. (2021) 13:621. doi: 10.3390/nu13020621, 33672996 PMC7918910

