# Supplementary material for: Joint effects of diabetes and time-weighted average multidimensional nutritional indices on mortality in patients undergoing peritoneal dialysis: a landmark analysis
Source: Front Nutr. 2026 Sep 18;13:1968835. doi: 10.3389/fnut.2026.1968835 (PMC13630681; doi:10.3389/fnut.2026.1968835)
Supplement: Supplementary file 1 [file Table_1.DOCX]

**Supplementary Material**

**Table S1. Scoring criteria for the Controlling Nutritional Status (CONUT) score.**

**Table S2. Missing data of the study participants**

**Table S3. Comparison of baseline characteristics between included and excluded patients**

**Table S4.** Frequency distribution of concurrent laboratory measurements for TWA nutritional indices calculation during the 12-month landmark period

**Table S5.** Sensitivity analysis: Comparison of predictive performance between baseline and time-weighted nutritional indices

**Table S1. Scoring criteria for the Controlling Nutritional Status (CONUT) score.**

| **Parameter** | **Normal** | **Light malnutrition** | **Moderate malnutrition** | **Severe malnutrition** |
| --- | --- | --- | --- | --- |
| **Serum albumin** |  |  |  |  |
| **Level (g/L)** | **>= 35.0** | **30.0 – 34.9** | **25.0 – 29.9** | **< 25.0** |
| **Score** | **0** | **2** | **4** | **6** |
| **Total cholesterol** |  |  |  |  |
| **Level (mg/dL)** | **>= 180** | **140 – 179** | **100 – 139** | **< 100** |
| **Score** | **0** | **1** | **2** | **3** |
| **Total lymphocyte CONUT** |  |  |  |  |
| **CONUT (/mm3)** | **>= 1,600** | **1,200 – 1,599** | **800 – 1,199** | **< 800** |
| **Score** | **0** | **1** | **2** | **3** |

**Table S2. Missing data of the study participants**

| **Variable** | **Number of missing values** | **Missing rate (%)** |
| --- | --- | --- |
| eGFR | 6 | 2.6 |

**Table S3.** Comparison of baseline characteristics between included and excluded patients

| **Characteristics** | **Total (n = 518)** | **Excluded (n = 289)** | **Included (n = 229)** | **P value** |
| --- | --- | --- | --- | --- |
| Age, years | 49.1 ± 14.3 | 49.9 ± 15.1 | 48.1 ± 13.2 | 0.156 |
| Male, n (%) | 322 (62.3%) | 183 (63.5%) | 139 (60.7%) | 0.671 |
| Smoking, n (%) | 90 (18.9%) | 55 (22.4%) | 35 (15.3%) | 0.049 |
| Alcohol consumption, n (%) | 52 (10.9%) | 39 (15.9%) | 13 (5.7%) | <0.001 |
| **Comorbidities** |  |  |  |  |
| Hypertension, n (%) | 436 (91.8%) | 225 (91.5%) | 211 (92.1%) | 0.788 |
| Heart disease, n (%) | 165 (34.7%) | 85 (34.6%) | 80 (34.9%) | 0.930 |
| Stroke, n (%) | 64 (13.5%) | 34 (13.8%) | 30 (13.1%) | 0.818 |
| **Clinical & Laboratory Measures** |  |  |  |  |
| BMI, kg/m² | 23.8 ± 4.3 | 23.6 ± 4.7 | 23.9 ± 4.0 | 0.451 |
| Hemoglobin, g/L | 86.7 ± 20.3 | 86.6 ± 20.7 | 86.9 ± 20.0 | 0.890 |
| Albumin, g/L | 35.6 ± 5.8 | 35.2 ± 6.1 | 36.0 ± 5.4 | 0.156 |
| Total cholesterol, mmol/L | 4.5 ± 1.2 | 4.5 ± 1.3 | 4.5 ± 1.2 | 0.877 |
| Triglycerides, mmol/L | 1.8 ± 1.1 | 1.8 ± 1.1 | 1.8 ± 1.0 | 0.654 |
| Lymphocyte count, ×10⁹/L | 1.2 ± 0.5 | 1.2 ± 0.5 | 1.2 ± 0.5 | 0.593 |
| Platelet count, ×10⁹/L | 188.2 ± 75.4 | 189.8 ± 81.3 | 186.6 ± 69.0 | 0.664 |
| eGFR, mL/min/1.73m² | 5.9 (4.4–7.9) | 5.9 (4.3–8.2) | 5.8 (4.5–7.6) | 0.695 |
| **Medications** |  |  |  |  |
| ARB/ACEI, n (%) | 130 (25.1%) | 69 (23.9%) | 61 (26.6%) | 0.471 |
| CCB, n (%) | 334 (64.5%) | 173 (59.9%) | 161 (70.3%) | 0.014 |
| Diuretic, n (%) | 53 (10.2%) | 34 (11.8%) | 19 (8.3%) | 0.196 |
| Beta-blockers, n (%) | 227 (43.8%) | 121 (41.9%) | 106 (46.3%) | 0.314 |

Abbreviations: BMI, body mass index; eGFR, estimated glomerular filtration rate; ARB/ACEI, angiotensin receptor blockers/angiotensin-converting enzyme inhibitors; CCB, calcium channel blockers.

**Table S4.** Frequency distribution of concurrent laboratory measurements for TWA nutritional indices calculation during the 12-month landmark period

| **Number of Measurements** | **Number of Patients (N = 229)** | **Percentage (%)** |
| --- | --- | --- |
| 2 | 56 | 24.45 |
| 3 | 95 | 41.48 |
| 4 | 52 | 22.71 |
| 5 | 21 | 9.17 |
| 6 | 4 | 1.75 |
| 7 | 1 | 0.44 |
| Median (IQR) | 3 (2 - 4) | **-** |

**Table S5.** Sensitivity analysis: Comparison of predictive performance between baseline and time-weighted nutritional indices

| **Indicator** | | **C-index (Baseline)** | | **C-index (TWA)** | **Δ C-index** | | **P value** | |
| --- | --- | --- | --- | --- | --- | --- | --- | --- |
| All-cause mortality | |  | |  |  | |  | |
| PNI | | 0.789 (0.736–0.842) | | 0.808 (0.756–0.860) | 0.019 | | 0.090 | |
| CONUT | | 0.789 (0.734–0.844) | | 0.818 (0.766–0.869) | 0.029 | | 0.076 | |
| HALP | | 0.777 (0.720–0.835) | | 0.787 (0.731–0.843) | 0.010 | | 0.202 | |
| Cardiovascular mortality | |  | |  |  | |  | |
| PNI | 0.832 (0.774–0.891) | | 0.827 (0.768–0.886) | | | -0.005 | 0.695 |  |
| CONUT | 0.819 (0.755–0.884) | | 0.838 (0.777–0.899) | | | 0.018 | 0.359 |  |
| HALP | 0.824 (0.755–0.894) | | 0.811 (0.736–0.886) | | | -0.014 | 0.195 |  |

Abbreviations: PNI, Prognostic Nutritional Index; CONUT, Controlling Nutritional Status score; HALP, Hemoglobin, Albumin, Lymphocyte, Platelet score; TWA, time-weighted average.

Notes:Δ C-index represents the difference between TWA-based and baseline models. P values were calculated using the compare C method.
